# Age-related declines in immune response in a wild mammal are unrelated to immune cell telomere length

**DOI:** 10.1098/rspb.2015.2949

**Published:** 2016-02-24

**Authors:** Christopher Beirne, Laura Waring, Robbie A. McDonald, Richard Delahay, Andrew Young

**Affiliations:** 1 Centre for Ecology and Conservation, University of Exeter, Penryn Campus, Cornwall TR10 9EZ, UK; 2 National Wildlife Management Centre, Animal and Plant Health Agency, Woodchester Park, Gloucestershire GL10 3UJ, UK; 3 Environment and Sustainability Institute, University of Exeter, Penryn Campus, Cornwall TR10 9EZ, UK

**Keywords:** eco-immunology, immune-competence, ageing, immunity, multivariate, wild population

## Abstract

Senescence has been hypothesized to arise in part from age-related declines in immune performance, but the patterns and drivers of within-individual age-related changes in immunity remain virtually unexplored in natural populations. Here, using a long-term epidemiological study of wild European badgers (*Meles meles*), we (i) present evidence of a within-individual age-related decline in the response of a key immune-signalling cytokine, interferon-gamma (IFN*γ*), to *ex vivo* lymphocyte stimulation, and (ii) investigate three putative drivers of individual variation in the rate of this decline (sex, disease and immune cell telomere length; ICTL). That the within-individual rate of age-related decline markedly exceeded that at the population level suggests that individuals with weaker IFN*γ* responses are selectively lost from this population. IFN*γ* responses appeared to decrease with the progression of bovine tuberculosis infection (independent of age) and were weaker among males than females. However, neither sex nor disease influenced the rate of age-related decline in IFN*γ* response. Similarly, while ICTL also declines with age, variation in ICTL predicted neither among- nor within-individual variation in IFN*γ* response. Our findings provide evidence of within-individual age-related declines in immune performance in a wild mammal and highlight the likely complexity of the mechanisms that generate them.

## 1. Introduction

Late-life declines in survival and reproductive success are pervasive in wild populations [1], but the physiological changes that give rise to such declines remain poorly understood. One key mechanism that has been hypothesized to contribute to organismal senescence is immunosenescence: declines in immune system function with advancing age, which increase susceptibility to infection and disease [2,3]. To date, the vast majority of immunosenescence research has focused on humans and laboratory organisms (e.g. [2]), with just a handful of principally cross-sectional studies conducted on wild populations (e.g. [4,5]; but see [6]). As such, the patterns and drivers of within-individual age-related changes in components of immunity remain virtually unexplored in natural populations under ecologically realistic conditions.

There is growing recognition that individual variation in cytokine production may play a major role in explaining the natural variation in immunocompetence observed in the wild [7]. Cytokines are the major signalling molecules of the immune system [7], with tight regulation of cytokine production being vital for the activation and propagation of appropriate immune responses [8]. Biomedical research has implicated age-related changes in pro-inflammatory cytokine production in contributing to immunosenescent declines [9]. First, a chronic increase in baseline pro-inflammatory cytokine concentration in the absence of an overt infection is known to be a risk factor for mortality and morbidity in elderly humans (a condition termed ‘inflammaging’ [10]). Second, research in laboratory mammals and humans has documented a general shift with increasing organism age from pro-inflammatory to anti-inflammatory cytokine production in response to immune stimulation [11]. Although such shifts are not ubiquitously detected [12], they have been suggested to contribute to the age-related weakening of cell-mediated immunity and the corresponding increase in disease susceptibility in elderly individuals [11]. Whether such age-related declines in pro-inflammatory cytokine responses to immune stimulation also occur in wild vertebrate populations is unknown, as are the factors that generate individual variation in the rate of age-related declines in such responses.

Life-history theory predicts that differences between males and females in their allocation of resources to costly activities such as reproduction and growth at the expense of immunity could drive sex-specific variation in immune defences [13] and sex differences in rates of immunosenescence [14]. The probability of acquiring and severity of progression of an infection are frequently higher in males than females [15]. It has also been suggested that sex differences in longevity and infection could be underpinned by slower immunosenescence in females than males [16]. Infection and disease themselves could also influence the rate of immunosenescence; accumulated tissue damage arising from inflammation associated with early-life or chronic infection, for example, may increase rates of immunosenescence [17,18]. As such, longitudinal within-individual assessments of age-related changes in cytokine production for individuals of known sex and disease status are now needed, to advance our understanding of the drivers of natural variation in cytokine responses.

Insight into the mechanisms that lead to immunosenescence may also be gained by comparing the age-related changes in multiple components of the immune system, as they may (i) have causal impacts on one another [19], and/or (ii) be a product of common underlying mechanisms [20,21]. One immune character that may contribute to age-related declines in the strength of the pro-inflammatory cytokine response is immune cell (leucocyte) telomere length (ICTL). ICTL is an important immune parameter that has previously been used as a biomarker of immunosenescence (e.g. [22]). Telomeres are protective nucleoprotein complexes found at the end of all eukaryotic chromosomes consisting of tandem (TTAGGG)^n^ nucleotide repeat sequences. Both the rate at which telomeres shorten and absolute telomere length have now been found to predict whole-organism survival and longevity in wild populations of a variety of species [23]*.* Immune cell telomeres specifically are known to shorten with age in humans and other mammals [23–25], and short immune cell telomeres are predictive of age-related disease and death in humans [26] and disease status in a wild mammal [25]. It has been suggested that declines in ICTL (e.g. with age) could generate concomitant declines in the pro-inflammatory cytokine response, in part by constraining lymphocyte proliferation potential [27]. One might therefore predict positive covariance between these two immune traits, both among individuals and within individuals over time (e.g. as individuals age). Such positive within-individual covariance might also be predicted if the age-related declines documented to date in both immune traits are the product of a common underlying mechanism [20]. Thus far, the few biomedical studies that have examined the relationship between ICTL and pro-inflammatory biomarkers have principally been cross-sectional in nature (precluding the examination of within-individual covariance between the two traits; e.g. [27,28]) and have yielded mixed results. Whether the within-individual age-related changes in these traits are positively associated in natural populations has yet to be investigated.

Here, we take advantage of a long-term epidemiological study of wild European badgers (*Meles meles*), to investigate the patterns and drivers of age-related variation in the production of the pro-inflammatory cytokine interferon-gamma (IFN*γ*) following immune stimulation. Production of IFN*γ* is important as it has a vital role in the regulation of both innate and adaptive immunity [7]. Its functions include the induction of antiviral enzymes, priming of macrophages and influencing leucocyte movement [7]. Biomedical research has suggested that IFN*γ* production in response to immune stimulation decreases with age in senescent cells both *in vitro* [29] and *ex vivo* in elderly humans (e.g. [30]). However, age-related declines in the IFN*γ* response are by no means ubiquitous (e.g. [31]); variation that may be attributable to differences between the strains of model organism employed and/or the principally cross-sectional nature of these laboratory studies. Whether such patterns extend to natural vertebrate populations is unknown. Here, we provide the first investigation of the patterns and drivers of age-related changes in IFN*γ* production in a natural population, using a unique longitudinal dataset of repeated *ex vivo* assessments of IFN*γ* production in response to immune stimulation for 295 wild badgers of known age, sex and bovine tuberculosis (bTB) infection status (960 measures over a 4-year period).

Specifically, we determine (i) if there is a within-individual age-related change in the magnitude of the pro-inflammatory cytokine response (see below for definition); (ii) if sex and/or disease (bTB infection status) influence the rate of within-individual age-related change in the pro-inflammatory cytokine response. Badgers that test positive for bTB in our study population show higher mortality rates [32], and males have a higher susceptibility both to infection and infection-related mortality than females [33]. Finally, as we have previously shown that within-individual age-related declines in ICTL occur in this population [25], and on the basis of the putative mechanisms outlined above for causal links between ICTL and the strength of the pro-inflammatory cytokine response, we investigate whether these two immune traits are positively associated by testing the following two predictions: (iii) that individuals that show greater pro-inflammatory cytokine responses also show longer average ICTLs (i.e. positive *among*-individual covariance between the two immune traits); and (iv) that within-individual variation in the pro-inflammatory cytokine response is positively correlated with within-individual variation in ICTL (i.e. positive *within*-individual covariance). In all cases, the pro-inflammatory cytokine response is defined as the magnitude of IFN*γ* production by whole blood cultured with a mitogen (pokeweed) known to non-specifically stimulate the proliferation of T- and B-cells [34]. Non-specific stimuli are thought to capture the responsiveness of an individual's immune system to a whole spectrum of pathogens better than antigen-specific stimuli (the responses to which are expected to relate more to one particular pathogen and its infection history [35]).

## 2. Material and methods

### (a) Study population

All badgers were captured as part of a long-term field study at Woodchester Park, Gloucestershire (UK), covering 11 km^2^ of woodland and surrounding farmland. The resident badger population has been subject to continuous ecological and epidemiological monitoring since the mid-1970s. In this high-density population, badgers live in social groups of up to 33 individuals occupying a common territory [36]. Badgers were trapped for two nights at all setts currently in use, four times per year. All badgers were anaesthetized and identified from unique alpha-numeric tattoos administered at their first capture. At every capture, the date, location, social group, sex, body mass (to nearest 100 g), body length and age class (juveniles less than 1 year, adult more than or equal to 1) of the captured individual were recorded. Captures of individuals of unknown age (those that were not identified as juveniles at their first capture event) were excluded from this analysis. For individuals that had been captured in their first year of life, age was defined as the number of days elapsed since 20 February in their first year of capture (reflecting the mid-February peak in births), as exact dates of birth cannot be readily determined [37]. All individuals captured were between 0.2 and 11.6 years of age. This study uses data from the 960 captures of 295 known-age individuals from 25 social groups between January 2010 and October 2013, when the following IFN*γ* response assay and bTB diagnostic tests were conducted for all individuals.

### (b) Interferon-gamma response to immune stimulation

Heparinized whole blood (4 ml adults; 2 ml juveniles) was collected from all captured individuals and used for the determination of IFN*γ* production in response to stimulation with pokeweed mitogen (PWM; a non-specific and generalized activator of T- and B-cells [34]) via an enzyme-linked immunosorbent assay (ELISA; see [38,39] for assay development, validation and full protocol). While PWM stimulation targets T- and B-cells, the magnitude of IFN*γ* production in response to a standardized PWM stimulus also includes IFN*γ* secreted from other cell types recruited during the inflammatory cascade (e.g. neutrophils). IFN*γ* response magnitude was measured in optical density (OD) units. As all samples were assayed for their response to PWM in duplicate (on the same plate), the average magnitude of the IFN*γ* response was used in all analyses (mean ± s.d. for average stimulated values was 0.99 ± 0.64). The mean within-plate co-efficient of variation between these replicates was 16.2% (*n* = 960). Each sample was also assayed in duplicate (on the same plate) with RPMI Medium 1640 (Fisher) in place of PWM, as an un-stimulated control (mean ± s.d. for average un-stimulated values was 0.06 ± 0.03). This allowed the IFN*γ* PWM-response values for all samples to be baseline-corrected prior to analysis (by subtracting from them the un-stimulated control). This conservative approach should ensure that any detected effects on the IFN*γ* response are attributable to differences in the response to stimulation rather than differences in baseline IFN*γ* concentrations. The outcomes of all analyses were qualitatively similar, however, if this baseline correction was not applied (see electronic supplementary material, S1). There is also considerable interest in age-related variation in baseline IFN*γ* expression levels [10], however the assay was not sufficiently sensitive to detect meaningful variation in baseline levels and thus baseline levels are not considered further.

### (c) Age

To ensure that our statistical assessment of within-individual variation in IFN*γ* response with advancing age was not confounded by between-individual effects (e.g. the selective disappearance of animals over time and hence with advancing age), we applied a within-subject centring approach, following [40]. Variation in age was partitioned into (i) an individual's ‘mean age’ across all samples collected for that individual, and (ii) its ‘Δ age’, i.e. the offset of its age at the focal sampling point from its mean age, the effect of which will reflect *within*-individual changes in the IFN*γ* response with age.

### (d) Body condition

We tested for a relationship between body condition and IFN*γ* response in order to ensure that any age effects were not being driven by a relationship between cytokine production and age-related changes in body condition [41]. Body condition was estimated using the scaled mass index (SMI: [42]) to account for the fact that variance in body mass increases with body length and that males are on average larger than females. The SMI has been found to capture variation in fat and protein reserves more effectively than traditional residual body condition indices [42].

### (e) Disease status

An individual's bTB infection status was assessed at each capture event using a combination of two diagnostic tests: (i) STAT-PAK^®^ (Chembio Diagnostic Systems), a lateral-flow immunoassay assessing the presence of antibodies to bTB; and (ii) microbiological culture of clinical samples (i.e. sputum, faeces, urine and swabs of wounds or abscesses) to detect *Mycobacterium bovis* (the causative agent of bTB) (see [43] for methods and a discussion of the performance of both tests). We used a simplified version of the disease classification system discussed in [44]. Briefly, individuals were classed as ‘negative’ if they had never tested positive for bTB on either test, ‘positive’ if they had ever tested positive using the STAT-PAK^®^ (Chembio Diagnostic Systems, Inc.), and ‘excretor’ if *M. bovis* had ever been identified by culture. This commonly adopted one-way progressive system is an appropriate way to assign disease status, as the bTB tests used show high specificity but low sensitivity [43], such that positive results are likely correct but false-negatives must be expected. This, coupled with the fact that bTB is known to be a chronic disease of badgers, which can be encapsulated within the body for long periods before subsequently re-emerging [33], leaves it most appropriate to classify individuals that have ever tested positive as ‘positive’ throughout their subsequent lifetimes, even if they subsequently test negative. Some previous studies have also used information from a third bTB test when classifying disease states in this population: an IFN*γ*-based ELISA test that draws inference from the ratio of the IFN*γ* responses to *ex vivo* stimulation of whole blood with bovine and avian tuberculin [38]. We have not used this test in our bTB disease state classifications, so as to avoid the possibility of a spurious correlation arising between disease status and our focal response term (the generalized IFN*γ* response to PWM stimulation) as both use aspects of the IFN*γ* response. Our findings regarding the influence of disease status were unaffected, however, if we did incorporate the ELISA bTB test into the disease state classification process as employed elsewhere (electronic supplementary material, S1). Of the 960 captures in our dataset, 586 were classed as ‘negative’, 326 as ‘positive’ and 48 as ‘excretor’. A total of 50 individuals transitioned between disease categories during the study period, 40 transitioned from ‘negative’ to ‘positive’, four from ‘positive’ to ‘excretor’ and six from ‘negative’ to ‘excretor’.

### (f) Immune cell telomere length

Average ICTL data were available from a previous study of European badger ICTL dynamics conducted on our study population [25]. Briefly, a second whole blood sample (4 ml adults; 2 ml juveniles) was collected from all captured individuals for estimation of average ICTL between May 2012 and October 2013. ICTL was determined via a robust and repeatable relative qPCR approach then converted to an absolute telomere length measure (kb) using standard methods (see electronic supplementary material, S2, for comprehensive methods and assay validation details).

### (g) Univariate modelling

In order to determine if the IFN*γ* response (960 measures from 295 individuals) declines with increasing age, and whether sex and disease status influence the rate of any age-related decline, we implemented a multi-model inference approach [45] using linear mixed-models. A total of 29 *a priori* candidate models were defined containing additive effects of all candidate explanatory variables (mean age, Δ age, sex, bTB status and body condition) and all biologically relevant two-way interactions (see electronic supplementary material, S3, for full model table). Interactions with Δ age were used to test whether sex or disease status specifically impacted the rate of *within-individual* age-related change in the IFN*γ* response. The response term for all models, IFN*γ* response to PWM (OD units), was log_10_ transformed in order to normalize model residuals. Models were ranked using Akaike's information criterion correcting for small sample size (AICc) [45] then more complex models were removed from the analysis if a simpler nested version of that model attracted greater support (a lower AICc) [46]. Following such removals, the remaining models with some support (defined as ΔAICc < 6 from the best-supported model) were retained in the top model set. Support for the models in the top set are discussed in terms of their relative ‘weights' defined as the likelihood of a given model divided by the total likelihood of all candidate models in the top model set [45]. To account for repeated measures, heterogeneity between IFN*γ* plate runs, and variation in social group territory quality, we included ‘individual ID’, ‘plate ID’ and ‘social group’ as random intercept terms in all models. Goodness-of-fit was assessed through calculating conditional (total variance explained by the best-supported model) and marginal (variance explained by fixed effects alone) *R*^2^ formulations [47] and standard residual plot techniques.

### (h) Multivariate modelling

In order to determine if (i) individuals that show stronger average IFN*γ* responses also show longer average ICTLs (positive among-individual covariance between these two variables) and/or (ii) within-individual variation in the IFN*γ* response (for example, as individuals age) is positively correlated with within-individual variation in ICTL (positive within-individual covariance) we used a Bayesian mixed-model approach (package MCMCglmm, Markov chain Monte Carlo generalized linear mixed models; [48]), in R [49]. The advantage of this approach is that it allows examination of the posterior correlation (and its corresponding confidence interval) between the magnitudes of the IFN*γ* response and ICTL, while controlling for effects of both fixed (e.g. bTB status and sex) and random factors (e.g. assay plate) on both immune traits, which could influence the apparent relationship between the traits.

We fitted a bivariate response model with IFN*γ* response magnitude (960 observations from 295 individuals) and absolute ICTL (360 observations from 172 individuals) as response terms. Both traits were Gaussian distributed. IFN*γ* was modelled as a function of bTB status and sex (the top model as revealed by the univariate modelling process above with age effects removed). Absolute telomere length was modelled as a function of bTB status (the top model as revealed by the univariate modelling process presented in [25] with age effects removed). Plate was fitted as a heterogeneous random effect for each trait, and completely parametrized (co)variance matrices for the individual identity random effect were used to allow covariance between each trait. Inverse gamma priors were used with 350 000 iterations, a burn-in interval of 80 000 and a thinning interval of 500. Repeatability and posterior correlations for the among- and within-individual covariance between IFN*γ* production and ICTL were estimated according to the methodology outlined in [50]. Support for a relationship between the traits would be represented by a correlation estimate with 95% credible intervals not spanning zero. Age was not initially included as a predictor of either immune trait in the bivariate model, so as to allow for any correlated age-related variation in each trait to become manifest as covariance between the two traits. We then repeated the modelling process with the fixed effects of age included for both traits (partitioned age; mean age and Δ age), but as the inclusion of partitioned age did not alter the findings of the covariance analysis we do not discuss this secondary analysis further.

## 3. Results

### (a) Age-related declines in the pro-inflammatory cytokine response

**Figure 1.**
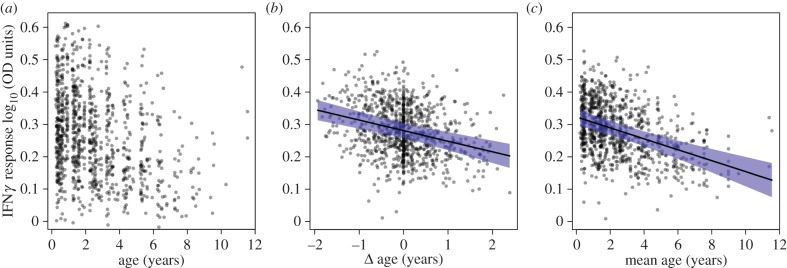
Relationship between age and IFN*γ* response magnitude. (*a*) Raw data plot of the relationship between IFN*γ* and un-partitioned age; (*b*) and (*c*) predicted relationship between IFN*γ* and Δ age (within-individual changes in age) and mean age (between-individual changes in age), respectively, from the top model in table 1. The grey points present raw data (*a*) or model residuals (*b*,*c*), black lines present model predictions and blue shaded areas present 95% confidence intervals.

Simply examining the relationship between the IFN*γ* response and un-partitioned age strongly suggests the occurrence of an age-related decline in pro-inflammatory cytokine production (figure 1*a*). After partitioning age to separate within- and among-individual effects, we found strong support for a within-individual decline in IFN*γ* response with advancing age (figure 1*b*), while controlling for the effects of sex, bTB status, assay plate and both individual and social group identities (figure 1*b*,*c* and table 1; see also electronic supplementary material, S3, for full model comparisons). The age-related decline in IFN*γ* response cannot be attributed to the presence of bTB-infected individuals in the dataset, as full statistical support for a within-individual age-related decline in the IFN*γ* response remained after the exclusion of all known-infected individuals (‘positive’ or ‘excretor’ disease classes; see electronic supplementary material, S4). That the within-individual decline in IFN*γ* response with age (the effect of Δ age; table 2) occurred at twice the rate of the among-individual decline in IFN*γ* response with age (the effect of mean age; table 2), with neither effect size overlapping the 95% confidence intervals of the other, suggests that individuals with weak IFN*γ* responses are being selectively lost from the population over time. We found no support for the rate of within-individual age-related change in IFN*γ* response accelerating or decelerating with increasing age (i.e. no statistical support for an interaction between Δ age and mean age; electronic supplementary material, S3).

**Table 1.** Results of univariate modelling of the factors influencing IFN*γ* response magnitude. Int = intercept; Δ age = within-individual age term; mean age = between-individual age term; bTB = bovine tuberculosis status (‘−ve’ = negative, ‘+ve’ = positive, ‘ex’ = excretor); cond = condition; d.f. = degrees of freedom; ΔAICc = deviation in AICc from the best-supported model; AW = adjusted model weight for models included in the top model set; ✓ = factors level included in the model with their corresponding contrasts adjacent to them; terms in italic denote the best-supported model. For unabridged model output, see electronic supplementary material, S3.

			sex		bTB						
int	Δ age	mean age	female	male	−ve	+ve	ex	cond	d.f.	ΔAICc	AW
*0.323*	−*0.033*	−*0.017*	✓	−*0.025*	✓	−*0.013*	−*0.057*		*10*	*0.00*	*0.67*
0.358	−0.032	−0.017			✓	−0.014	−0.062	−0.006	10	3.19	0.13
0.319	−0.036	−0.018	✓	−0.027					8	3.82	0.10
0.311	−0.033	−0.016			✓	−0.014	−0.060		9	3.83	0.10

**Table 2.** Coefficients and confidence intervals from the best-supported model in table 1. *β*-estimate = direction and magnitude of a parameters effect; s.e. = standard error; CI = confidence interval; and terms in brackets = reference level for factors (with addition levels shown below).

parameter (reference level)	*β*-estimate	s.e.	95% CI
Δ age	−0.033	0.006	−0.044 to −0.021
mean age	−0.017	0.003	−0.022 to −0.012
bTB status (negative)			
exposed	−0.013	0.010	−0.032 to 0.007
excretor	−0.057	0.021	−0.097 to −0.016
sex (female)			
male	−0.025	0.010	−0.045 to −0.005

### (b) Impacts of sex and disease status on the pro-inflammatory cytokine response

**Figure 2.**
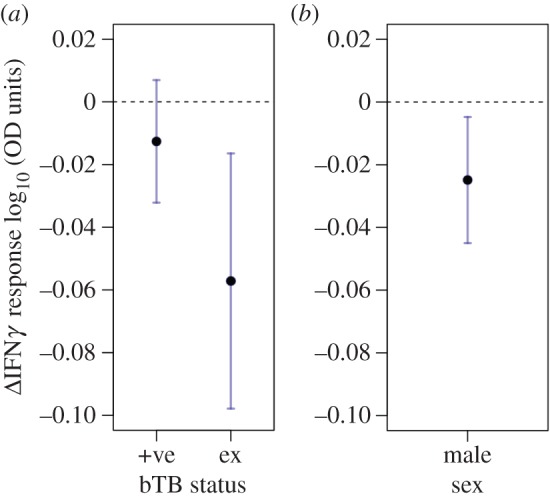
Sex and disease-associated variation in IFN*γ* response. (*a*) Predicted change in IFN*γ* response due to bTB infection status (dashed baseline = negative, ‘+ve’ = positive, ‘ex’ = excretor) from the top model in table 1, (*b*) predicted change in IFN*γ* in males in comparison to females (dashed baseline) from the top model in table 1. The black points present model predictions and the error bars present 95% confidence intervals.

We found strong support for an age-independent effect of bTB infection status on the IFN*γ* response, with ‘excretor’ classes having weaker IFN*γ* responses than both ‘negative’ and ‘positive’ classes (figure 2*a* and table 2). However, we found no evidence that disease status influenced the within-individual rate of decline in IFN*γ* response with age (electronic supplementary material, S3). We also found support for males having weaker IFN*γ* responses than females (figure 2*b*). However, again, there was no evidence to suggest that the sexes differed in their within-individual rate of decline in IFN*γ* response with age (i.e. no support for an interaction between sex and Δ age; electronic supplementary material, S3). We found very weak support for a previously reported link between an individual's current body condition and the pro-inflammatory cytokine response (table 1; [41]). The best-supported univariate model explained 59% of the total variation in the IFN*γ* response, with the fixed effects accounting for 12%, assay plate (between-plate variation) for 25%, individual identity for 19% and social group for 3%.

### (c) Covariance between the pro-inflammatory cytokine response and immune cell telomere length

Both IFN*γ* response (Repeatability_IFN*γ*_: 0.57; 95% CI: 0.50 to 0.61) and average ICTL (Repeatability_ICTL_: 0.42; 95% CI: 0.30 to 0.55) showed repeatable among-individual differences through time, and both traits showed clear within-individual declines with age (IFN*γ* response: figure 1*b* and table 1; average ICTL: [25]). Despite this, the two traits appear to be independent of one another, as we found no support for the existence of either among-individual covariance (*r*_ind_: 0.02; 95% CI: −0.14 to 0.20) or within-individual covariance (*r*_e_: −0.02; 95% CI:−0.10 to 0.15) between the two immune traits (see electronic supplementary material, S5, for full model table).

## 4. Discussion

The results presented here strongly suggest that within-individual age-related declines in pro-inflammatory cytokine production occur in this wild population of mammals. By coupling the longitudinal repeat-sampling of individuals with an age-partitioning approach, we have avoided the confounding complications that are expected to arise in cross-sectional studies from selective disappearance effects. That the within-individual rate of age-related decline in the IFN*γ* response was twice the among-individual rate of decline suggests that individuals with weaker IFN*γ* responses are being selectively lost from this study population (via mortality and/or dispersal). As the study population has a high average annual recapture probability (0.80; [51]) and dispersal events in to and out of the population are rare [52], it is more likely that these patterns reflect a survival cost entailed in having weaker IFN*γ* responses, rather than the differential dispersal of individuals in this state. We found strong evidence to suggest that the progression of bTB infection to the ‘excretor’ stage is associated with a reduction in the IFN*γ* response, and evidence that male badgers have a weaker IFN*γ* response than females. Neither sex nor disease status, however, predicted the rate of the within-individual age-related decline in the IFN*γ* response. Despite putative mechanistic links between ICTL and the pro-inflammatory cytokine response, and the existence of age-related declines and repeatable individual variation in both immune traits, we found no evidence that (i) individuals with longer ICTLs mounted stronger IFN*γ* responses, or (ii) that within-individual variation in ICTL was positively correlated with within-individual variation in the IFN*γ* response. Below, we discuss the potential mechanisms underpinning the observed declines in these immune traits with age and their implications for our understanding of senescence in natural populations.

We used an *ex vivo* lymphocyte stimulation assay in order to measure how the production of a key pro-inflammatory cytokine changes with increasing age. Our findings suggest that there is a within-individual decline with increasing age in the strength of the pro-inflammatory IFN*γ* response to this form of immune stimulation (using a non-specific mitogen; PWM). The decline documented here is consistent with previous work in humans and laboratory model organisms demonstrating that weakening IFN*γ* responses with age contribute to a general shift from pro-inflammatory to anti-inflammatory cytokine responses in later life (e.g. [53]). Age-related changes in cytokine expression profiles have also been implicated in mediating thymic involution (the gradual shrinking of the thymus with age), which is thought to be one of the major contributing factors to the immunosenescence phenotype [54]. It is important to note, however, that as only one pro-inflammatory immune marker was assayed, we cannot disentangle immune impairment from immune remodelling (whereby decreases in IFN*γ* production could be compensated, for example, by the upregulation of other pro-inflammatory cytokines). Likewise, the observed age-related declines in the IFN*γ* response could occur in the absence of any net shift towards an anti-inflammatory immune response (as in [55]). It is also possible that a reduction in the IFN*γ* response with age could be adaptive, rather than pathological, particularly if it compensated for the age-related increase in baseline inflammatory cytokine levels that have been documented, for example, in human populations (‘inflammaging’ [10]). We also make the implicit assumption here that a larger cytokine response equates to a more effective immune response, which may not necessarily be the case [7]; in several disease states exposure to an excess of cytokines can lead to tissue damage and death [56]. That said, our findings do suggest that weak IFN*γ* responses may carry a cost, as individuals with weaker IFN*γ* responses appear to be selectively disappearing from the population (see above). While the mechanisms giving rise to this apparent selective disappearance are unknown, they do not appear to involve a clear effect on body condition, as there was only weak support for a link between body condition and IFN*γ* response. It may be that individuals with weak IFN*γ* responses are more susceptible to infection and/or the more rapid progression of disease (see below).

We found evidence suggesting that males on average show weaker IFN*γ* responses than females. This is consistent with previous work demonstrating that male badgers also show weaker IFN*γ* responses to bTB antigen-specific lymphocyte stimulation [57], and might explain why males show higher susceptibility to, and reduced survival during the progression of, bTB in this species [33,58]. Sex differences in a variety of immune parameters appear widespread in vertebrates [59] and have been hypothesized to arise from male-biased mortality reducing selection for robust immune defences in males relative to females [59]. Mechanistically, the observed sex difference in cytokine production could arise from the immunosuppressive effects of testosterone in males [59], as circulating testosterone levels have been known to be inversely correlated with plasma cytokine levels [56].

Our analyses also highlighted an association between bTB disease progression and the magnitude of the IFN*γ* response, whereby individuals classed as ‘excretor’ showed weaker IFN*γ* responses than both disease ‘negative’ and ‘positive’ individuals. This pattern is compatible with our current understanding of how tuberculosis infection modulates the pro-/anti-inflammatory immune response in humans: *in vivo* and *in vitro* studies have shown that active tuberculosis infection can result in reduced pro-inflammatory responses [60,61]. Using observational datasets such as ours, however, it is near-impossible to distinguish whether the relationship observed here reflects a negative effect of disease progression on the pro-inflammatory response, or individuals with weak pro-inflammatory responses being predisposed to disease progression (a relationship that could account, at least in part, for the apparent selective disappearance of individuals with low IFN*γ* responses). That bTB ‘positive’ and ‘negative’ individuals did not differ in the magnitudes of their IFN*γ* responses does nevertheless suggest that, for bTB at least, the strength of the IFN*γ* response is more closely linked to disease progression than initial infection.

While both ICTL and the magnitude of IFN*γ* response show evidence of within-individual declines with age, repeatable individual differences (see also [25]) and links with selective disappearance (see also [25]), we found no evidence of within- or among-individual covariance between these two immune traits. This suggests that age-related declines in the two traits may be occurring independently, lending rare support from natural populations to the view that multiple mechanisms likely underpin age-related declines in different immune parameters [21]. As our analyses did not specifically investigate the patterns of covariance between the within-individual rate of change with age in one immune metric and the absolute values of the other, it remains conceivable that individuals with a stronger IFN*γ* response show higher rates of telomere attrition. However, our analyses do suggest that any such effect is not so strong as to have left individuals with consistently higher IFN*γ* responses with reduced mean ICTL. The hypothesized causal links between ICTL and the IFN*γ* response could be more complex than we had initially envisaged. While short immune cell telomeres could constrain the pro-inflammatory cytokine response (potentially generating positive covariance between the two traits; see Introduction; [27]), individuals with a strong pro-inflammatory phenotype could also experience elevated levels of immune cell turnover and/or oxidative stress, both of which can reduce ICTL (potentially generating negative covariance between the two traits). It is therefore possible that both processes are occurring concurrently, precluding the detection of a clear directional relationship between the two traits. It is also possible that our measures of average ICTL and the IFN*γ* response are too coarse to allow the detection of a relationship between the two traits. ICTL was estimated from a diverse population of immune cell subtypes (all leucocytes), which likely vary in the extent of their contribution to the IFN*γ* response to immune stimulation. Similarly, the IFN*γ* response was estimated through a whole-blood ELISA in which the contributions of different immune cell subtypes to the observed response cannot be ascertained. The application of more advanced methods to estimate immune cell subtype-specific telomere lengths (e.g. [62]) and relate these to subtype-specific cytokine production dynamics might therefore be necessary to detect the predicted relationships between ICTL and the IFN*γ* response.

Our findings demonstrate for the first time that within-individual age-related declines in the pro-inflammatory cytokine response occur in a natural vertebrate population. Furthermore, these declines appear to occur independently of immune cell telomere dynamics; one putative proximate mechanism influencing immunosenescent declines [25]. While we recognize that the measurement of a small number of immune biomarkers will never capture the complex and multifaceted immunosenescent phenotype, our work adds to a growing body of evidence suggesting that the reduced effectiveness of the immune system at advanced ages widely observed in humans and laboratory organisms does generalize to wild vertebrate populations. Further work specifically quantifying how these and other immune parameters contribute to an individual's susceptibility to infection, disease and mortality appears crucial in order to fully understand the fitness consequences and evolutionary implications of age-related declines in immune system capability.

## Supplementary Material

Electronic supplementary material

## Data accessibility

Data available from the Dryad Digital Repository: http://doi.org/10.5061/dryad.tj5f0.
